# Mouldable Collagen–Tricalciumphosphate Is a Safe Carrier for Local Antibiotics—Short-Term Results in Revision Hip Arthroplasty

**DOI:** 10.3390/antibiotics13060510

**Published:** 2024-05-30

**Authors:** Yannik Hanusrichter, Carsten Gebert, Sven Frieler, Burkhard Moellenbeck, Marcel Dudda, Martin Wessling, Christoph Theil

**Affiliations:** 1 Department of Tumour Orthopaedics and Revision Arthroplasty, Orthopaedic Hospital Volmarstein, 58300 Wetter, Germany; 2 Center for Musculoskeletal Surgery, University Hospital of Essen, 45147 Essen, Germany; 3 Department of Orthopedics and Tumor Orthopedics, Muenster University Hospital, 48149 Muenster, Germany; 4 Department of Trauma and Orthopedic Surgery, BG University Hospital Bergmannsheil, Ruhr University Bochum, 44879 Bochum, Germany; 5 Department of Trauma Surgery, University Hospital Essen, 45147 Essen, Germany; 6 Department of Orthopedics and Trauma Surgery, BG-Klinikum Duisburg, University Duisburg-Essen, 47249 Duisburg, Germany

**Keywords:** revision arthroplasty, local antibiotics, PJI, periprosthetic joint infection, revision hip arthroplasty, tricalciumphosphate

## Abstract

Background: Improving local antibiotic delivery is a promising approach to improve infection control and potentially shorten systemic treatment in periprosthetic joint infection (PJI). This study investigates the use of an antibiotic-loaded, mouldable collagen–tricalciumphosphate composite in treatment of hip PJI. Methods: 124 application cases in 79 patients were included from a referral centre; systemic adverse infects, local complications, and infection control were analysed. Results: In most cases, either vancomycin or meropenem were used. Pathogens were previously known in 82 (66%) cases with polymicrobial infection in 20 (25%) patients. There were no cases of hypercalcaemia. Acute kidney injure was present in 14 (11%) cases. Chronic kidney failure persisted in two cases. During a mean follow-up of 12 (SD 9.3; range 3–35) months, implant survival was achieved in 73 (92%) patients; revision due to PJI was performed in 19 cases. Conclusion: Mouldable collagen–tricalciumphosphate composite bone substitute as a local antibiotic carrier in revision hip arthroplasty appears to be a valid option for local antibiotic delivery without systemic complications. Implant survival of 92% supports the hypothesis that local antibiotic therapy is an important component in the treatment of PJI.

## 1. Introduction

PJI is a devastating complication following total hip arthroplasty (THA). The rate of PJI is reported to be around 1% for primary implantations [1] and is among the leading cause for revision THA [2]. Furthermore, in revision THA, the risk of PJI can be as high as 15%, particularly if the initial cause for revision was PJI [3]. Patients with large bone defects requiring metallic augmentation or megaprosthetic reconstruction appear to be at greatest risk for PJI. In these complex cases, PJI may occur in more than 20% [4,5,6].

The optimal management of PJI depends on a number of factors such as the acuteness of infection, host status, known organism and antibiotic susceptibility, and integrity of the implant [7]. For chronic infections, particularly if large bone defects are present or the infection is caused by highly resistant, difficult-to-treat organisms, a two- or multi-stage approach is commonly used [3,5,6]. During the first stage, all infected tissues and all foreign material are removed and irrigation is performed. After wound healing is sufficient and there are no signs of a persisting infection, second-stage reimplantation of a revision implant is performed.

While many factors may contribute to the success of staged management of hip PJI, the cornerstones include complete debridement and implant removal followed by several weeks of systemic antibiotic treatment. However, the role of local antibiotic carriers and bone substitutes are still to be determined [8,9].

While it can be difficult to achieve adequate antibiotic concentrations in the joint itself via the application of systemic antibiotics, it is unclear which method of local antibiotic application achieves the best results in terms of initial burst and long-term liberation [9]. Traditionally, a polymethylmethacrylate spacer is used as part of a staged approach, as it also provides local stability and fills the dead space. However, antibiotic elution is limited to around 2–3 weeks, after which the spacer might be colonized itself and the spacer construct may lead to mechanical complications [6,8]. Additionally, intramedullary spacer stabilization requires a minimum amount of bone stock, which might be highly insufficient in re-revisions. More recently, calcium-based antibiotic carriers have been introduced, which can be combined with various antibiotics and have shown favourable elution kinetics with adequate release over six weeks and longer with relatively few adverse effects [10,11,12].

Additionally, particularly on the acetabular side, there may be cavitary bone defects as well as screw holes left following removal of the previous infected THA; β-tricalciumphosphate (ß-TCP) can act as an introductory structure to induce the restauration of acetabular bone stock [13]. However, there is concern that bone substitutes might become infected if used in cases of hip PJI [14]. Antibiotic loading of synthetic bone substitute may overcome this concern and, furthermore, improve local antimicrobial efficacy [15].

In particular, composite materials [16] containing a resorbable synthetic bone graft and collagen offer the advantage of sufficient antibiotic loading capacity. Furthermore, antibiotic elution appears to be superior [17].

**Figure 1:**
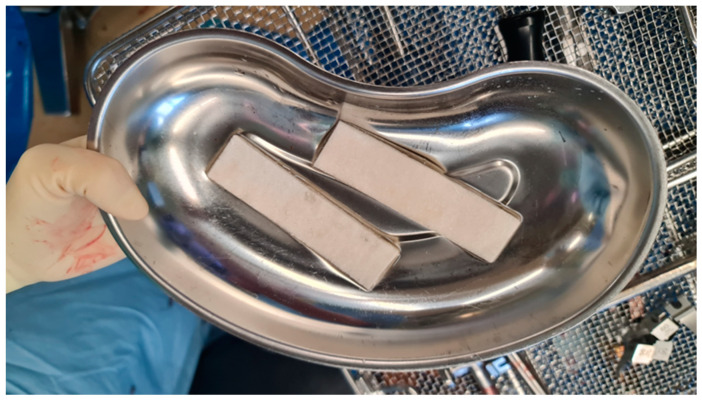
Antibiotic-loaded, mouldable ß-TCP collagen foam.

However, clinical studies on the use of such materials in hip PJI are limited by small sample sizes and heterogeneous use of different bone substitutes, which makes it difficult to determine appropriateness of their use in these complex cases [18,19]. To our knowledge, published articles regarding the usage of β-TCP collagen composite, as shown in Figure 1, are sparse and limited to case reports [20]. Its combination with antibiotic loading has not been studied in the treatment of hip PJI.

This study investigates the use of a β-TCP collagen composite bone substitute that was loaded with antibiotics for acetabular defects as part of the management of hip PJI in patients with extensive bone defects, focusing on short-term complications as well as infection control in a consecutive series of hip PJIs.

## 2. Operative Algorithm and Antibiotic Loading

Suitability for antibiotic-loaded ß-TCP (Cerasorb Foam^®^, Curasan AG, Frankfurt, Germany) was based on several parameters: (I) known preoperative pathogen, (II) extensive acetabular or femoral defects, and (III) subjective assessment as a difficult case due to secondary factors (e.g., multimorbid patients or critical soft tissue). The treatment approach (either DAIR, one-stage, or two-stage exchange) was based on the referral centre algorithm set by a multidisciplinary board, as well as published guidelines [21]. If either parameter was present, one- or two-antibiotic-loaded ß-TCP mouldable foams were used (12 × 12 × 4 mm). The collagen–tricalciumphosphate composite was manually loaded with specific antibiotics, depending on preoperative pathogen knowledge and resistance testing. For Vancomycin and Meropenem, 2 g was mixed with 10 mL aqua and applied to the foam; for other agents (e.g., voriconazole or gentamycin), an infectious disease specialist consultation was conducted preoperatively. If no pathogen was detected preoperatively vancomycin (+/− meropenem) was used. After a set exposure time of at least 10 min, the foam was moulded into the defect. The application process can be seen in the video added as a Supplementary Materials . All patients received systemic antibiotic application for a total treatment duration of 12 weeks in a standardized algorithm set by a multidisciplinary team. For the initial two weeks after each surgery, intravenous application was carried out, followed by oral therapy [21].

## 3. Results

In 15 cases, more than one agent was used, resulting in an application of at least two foams. The most used was vancomycin (*n* = 100, 80%), followed by meropenem (*n* = 37, 29%); the mean agent dosage was 2.4 (SD 1.4; range 0.14–4) grams. Detailed foam statistics are shown in Table 1. The causative pathogens were known preoperatively in 82 (66%) cases. A total of 20 (25%) patients suffered from a polymicrobial infection and 13 (16%) patients suffered from a culture-negative PJI. The pathogens are presented in Table 2.

While not the main focus of this study, we report the infection rates for completeness. During a mean follow-up period of 13 (SD 9.3; range 3–35) months, the all-cause revision rate was 24% (29/124). Revision due to persistent or recurrent PJI was performed in 17% (19/124) of cases; in nine cases, this was performed during the interval period in a two-stage exchange.

**Figure 2:**
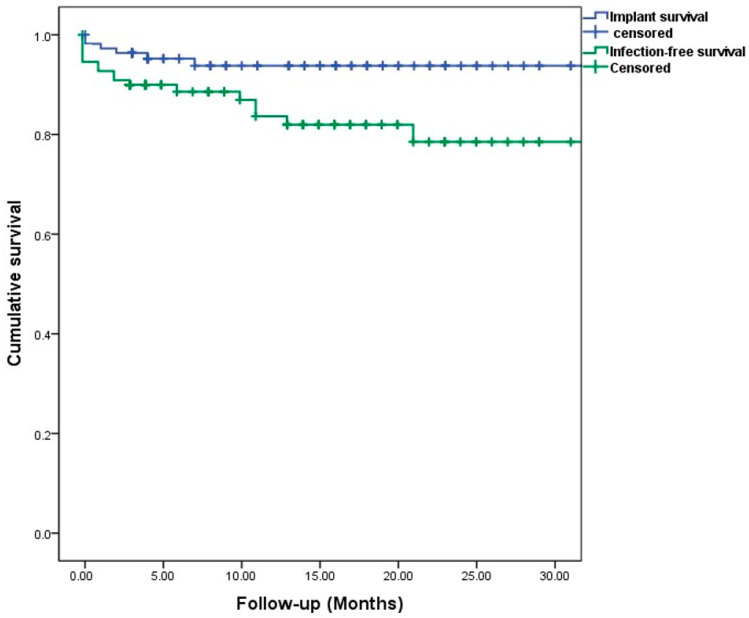
Patient-specific Kaplan–Meier survival estimates for implant and infection-free survival. Censored: follow-up endpoint, marked as a tick.

**Figure 3:**
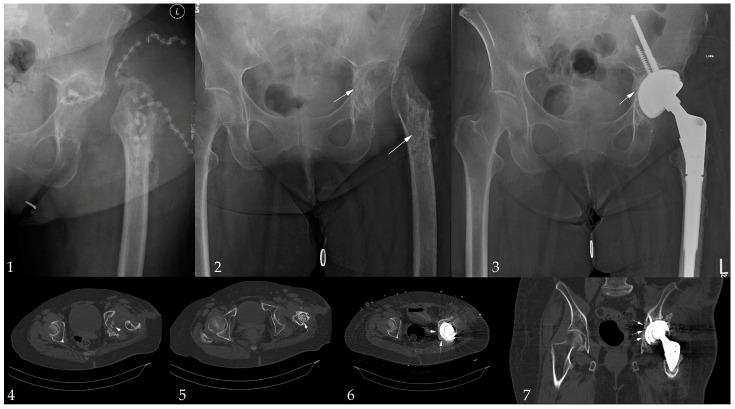
Exemplary case: 52-year-old female patient with multiple revision due PJI after THA in an initial femoral neck fracture presenting with a polybacterial PJI *(Staphylococcus haemolyticus, Staphylococcus caprae, Klebsiella pneumoniae Enterococcus faecalis)*. Image 1: the admission situation; Image 2: initial debridement; Image 3: final image after 1-year follow-up; Image 4, 5: acetabular and femoral ß-TCP application; and Image 6, 7: ß-TCP application during the implantation of an rTHA in a Paprosky IIC defect used for medial augmentation. Arrows mark the ß-TCP foams.

Implant survival following infection treatment was achieved in 73 (92%) patients at last follow-up, explantation due to PJI was conducted in two cases, an aseptic loosening or periprosthetic fracture occurred in four cases. The survival rates are shown in the Kaplan–Meier analysis (Figure 2). A total of ten (12%) patients were lost prior to follow-up; one patient died due to PJI shortly after explantation. Due to the heterogenous collective and, therefore, smaller sample size in each group, no significant risk factors could be analysed. An exemplary application in a two-stage setting is shown in Figure 3.

Postoperative serum creatinine levels during the first postoperative week showed a mean delta of 0.02 (SD 0.3; range −0.68–1.62). An increase of >0.3 mg/dL 48 h post operation was present in 14 (11%) cases. Doubling of the index level was detected in three (2%) cases during the first postoperative week. Chronic kidney failure (KDIGO G3aA1) persisted in two cases.

Postoperatively, the ionized serum calcium value showed a delta of −0.21 (SD 0.2; range −1.07–0.99); there was no newly diagnosed hypercalcemia present postoperatively.

**Figure 4:**
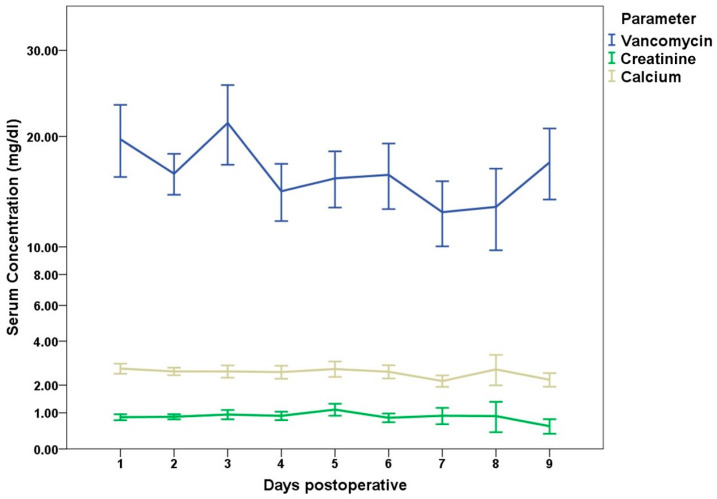
Absolute postoperative serum concentrations during the first 9 days post operation for Vancomycin, creatinine, and calcium.

Vancomycin, as a systemic therapy, was administered in 77 cases with a mean serum level of 16.1 (SD 7.1; range 1–37) mg/dL during the first week. In ten (12%) cases, the serum level remained elevated (>20 mg/dL) during the first week; in nine of these cases, TCP with vancomycin was used. The serum concentrations are shown in Figure 4.

## 4. Materials and Methods

In a single-centre study, we included all consecutive cases treated with antibiotic-loaded TCP between 2020 and 2022. The inclusion criteria included a revision total hip arthroplasty procedure due to PJI, regardless of the chosen treatment method (either DAIR, one-stage, or two-stage exchange). Diagnosis was based on the MSIS criteria. To detect all adverse outcomes, no exclusion criteria were defined. 

Ethical approval was obtained prior to the investigation from the local ethics committee (reference number 2022-021-f-S).

### 4.1. Patient Parameters

This study included 124 operations conducted in 79 patients at a high-volume referral centre between January 2020 and December 2022; during the same period, 310 hip revisions due to PJI were conducted. Antibiotic-loaded TCP was used in 40% of all cases, consisting of 12 DAIR, 12 one-stage, and 50 two-stage exchanges for both ex- and implantation. Basis parameters and application data are shown in Table 1.

### 4.2. Observed Indicators

Due to the novelty of the agent, analysis was conducted with consideration of systemic side effects, the complication rate, infection control, and short-term follow-up. Therefore, a minimum follow-up of 6 months was deemed sufficient. Postoperative serum creatinine, ionized calcium, and vancomycin (if administered systemically) were recorded three times during the first 7 postoperative days. Delta was calculated to the last preoperative value. The primary outcome parameter was revision due to ß-TCP-associated complications as well as persistent short-term PJI. Secondary outcome parameters were specified as postoperative systemic complications, defined as follows: Postoperative acute kidney failure was defined as an increasement of ≥0.3 mg/dL during the first 48 h or above >50% of the index level during the first seven days. Elevated vancomycin levels were defined as >20 mg/dL. Postoperative hypercalcemia was defined as elevated ionized serum levels above 5.2 mg/dL.

Data analysis was performed using the Statistical Package for Social Sciences Software (IBM SPSS Statistics Version 24, Chicago, IL, USA). Descriptive statistical results were recorded to describe comorbidities, complications, and previous procedures. A Shapiro–Wilk test was performed to determine non-normal/normal distribution. A T-test was used for parametric values and Mann–Whitney U for non-parametric values in univariate analysis. To determine risk factors, univariate and multivariate logistic regression analyses were conducted. The significance level was set at *p* < 0.05.

## 5. Discussion

This study is the first to evaluate the use of a custom-made, antibiotic-loaded composite collagen–tricalciumphosphate bone graft substitute as a bone void filler and antibiotic carrier in the treatment of hip PJI in 124 cases. Most patients had vancomycin or Meropenem added as a local antibiotic, based on the identified microorganism.

While short-term implant survival was achieved in 92% of patients at last follow-up, 17% of patients underwent unplanned revision surgery due to persisting/recurrent infection or wound complications. Furthermore, while 11% of cases had temporarily impaired kidney function, no patient had relevant serum calcium abnormalities.

While this is not a comparative study design, the study’s results suggest that excellent short-term infection control in multiple revised hip arthroplasties with severe bone loss and a complicated microorganism spectrum is possible with the use of a mouldable collagen–tricalciumphosphate composite as an antibiotic carrier. 

Traditionally, a two-stage approach for the management of chronic hip PJI comprises the use of an antibiotic-loaded PMMA spacer for local antibiotic delivery as well as dead space management and joint stability [3,22]. This approach can lead to long-term infection control in around 85% of patients in large studies from dedicated centres [3]. However, spacer complications, particularly dislocation and fracture, may occur during the interim period [22,23,24]. Patients with large bone defects appear to be at increased risk for such complications [24]. Furthermore, after release of the antibiotic dose, the spacer itself might cause recurrent infections [25]. While there is no consensus on the general use of local, calcium-based, degradable antibiotic carriers due to the lack of robust literature [9], there are promising results from individual centres. One study [19] investigated the use of a synthetic composite calcium sulphate/hydroxyapatite in complex hip revision arthroplasty in 49 patients. The authors used the composite material for acetabular augmentation in combination with calcium sulphate beads as an antibiotic carrier in the joint. They noted no acetabular loosening with adequate graft remodelling and integration in most cases. They reported a 24% reoperation rate in their complex and high-risk patient cohort with a 12% infection rate. However, no patients had to be revised for wound drainage. However, they did not report renal function or serum calcium results in their study. In conjunction with our findings, local antibiotic delivery and bone remodelling appear possible, even in extensive bone defects and after multiple failed revisions.

Acute kidney injury (AKIN) is a frequent complication in revision hip arthroplasty due to PJI, particularly in patients who have undergone first-stage implant removal and spacer insertion, and may occur in more than 30% of patients [26]. While the underlying reasons are multifactorial [27,28], exposure to potentially nephrotoxic, local, and systemic antibiotics in combination with postoperative anaemia appears to be a leading cause [26]. Due to the potentially variable release kinetic from the composite bone substitute that was manually loaded to act as an antibiotic carrier, patients in this study might be at an increased risk of AKIN. However, the risk of grade-one AKIN was only 11% and, therefore, considerably lower than in previous studies that evaluated staged revision for PJI [27]. The observation regarding the low systemic toxicity of calcium-based antibiotic carriers has also been discussed in a review article and a large study on their use in the treatment of chronic osteomyelitis [29,30]. However, systemic calcium exposure has been discussed when using calcium-based bone substitutes [31], although this appears to be particularly relevant in calcium-sulphate-based products. The collagen–tricalciumphosphate composite used in the present study was not associated with hypercalcemia. While claims regarding product safety must be used carefully, particularly when evaluation new techniques with the custom additions of antimicrobial drugs to bone substitutes, it appears that the approach chosen in this study is not associated with a relevant degree of systemic toxicity. Nonetheless, prospective studies are needed and patients need to be counselled regarding their individualized treatment approach, as severe complications are always possible in individual cases. 

Short-term implant survival was 92% at the last follow-up. However, 17% of the cases (*n* = 19)) underwent revision surgery for either recurrent or persisting infection, in 9 cases during the interval period. While wound leakage and conservative treatment has been described for the use of ceramic antibiotic carriers in the treatment of osteomyelitis [29], wound drainage after arthroplasty revision requires revision surgery, especially in the treatment of fulminant infections. Given that wound revision was not a major issue in previous studies [18,19] of hip revision surgery using a calcium-based (composite) bone graft as a local antibiotic carrier, it is unclear whether the cases of seroma with wound drainage were due to persistent/recurrent infection or if they could be related to the use of the composite bone graft. As a high number of patients had megaimplants on the femoral side or had undergone previous failed treatment for PJI; the higher revision rate and potential dead space must be considered and could potentially explain the rate of complications found [32,33,34]. One study on 19 repeat, staged THA revisions for PJI found the need for repeat revision due to recurrent/persisting infection in 42% of patients. In this context, with a megaprostheis reconstruction in 32 (40%) of the patients, the revision risk encountered appears to be acceptable, although patients must be advised that wound drainage with the need for revision could be aggravated if local antibiotic carriers using this composite bone substitute are used. Although several risk factors are known for re-revisions or persistent PJI, in this study, the authors are not able to validate these findings due to the smaller sample size [35].

Systemic vancomycin serum levels are known to fluctuate highly during the early postoperative period up to 10 days [36]. While there was a generally higher level during the first 3 postoperative days, the authors associate the increase between days 8–10 with better systemic application and a stabilised intravascular volume balance. Additionally, other topical applications with similar release kinetics have shown no influence on systemic concentrations and no adverse effects [37]. This is consistent with the results published by Colding-Rasmussen et al., who were able to report high local concentrations in the drain analysis without a significant increase in serum levels [38].

Local antibiotic carriers have been shown to be advantageous in multiple aspects, as they are (I) biodegradable, (II) osteoinductive, and (III) provide excellent long-term release kinetics [39]. While recommendations for local usage are mainly based on fracture-related infections, as published by Metsemakers et al., these can be transferred to PJI cases [40]. Steadmen et al. concluded a potential benefit for PJI cases, though they advised to analyse the application on a case-by-case basis [41]. While not the main focus of this study, the outcome seems to be slightly improved if local antibiotic carriers are added. Looking at comparable studies, McKee et al. already showed, in 2010, slightly better outcomes for bioabsorbable bone substitutes compared to PMMA in the treatment of chronic osteomyelitis [42]. This was further embedded in a meta-review by Shi et al. that evaluating the use of calcium sulphate carriers for chronic osteomyelitis, showing successful treatment in 92% of patients [43]. In PJI cases, Sakellariou et al. showed an improvement in infection eradication, although not statistically significant with local hydroxyapatite application (*p* = 0.192) [44]. However, it remains unclear which ceramic (either phosphate or sulphate) should be used. As phosphate inherits a prolonged resorption time, this can be beneficial to induce osseointegration in cases with reduced bone metabolism, as encountered in revision arthroplasty settings [29]. 

A secondary parameter to consider is the cost of each application. While this varies from country to country due to local conditions, we can report a price of around EUR 800 per unit in the centre used in this study. However, the potential reimbursement in the healthcare system, possible due to its classification as an allogenic ceramic bone graft, makes the foam cost-effective to use.

While this is a large, single-centre study that managed to include a high number of cases in a fairly short time period using a distinct and uniform diagnostic and treatment algorithm, it has several limitations: Firstly, the study population is extremely heterogenous, as all PJI treatment options (DAIR, one-stage, and two-stage exchanges) have been included; therefore, the results should be interpreted with caution in each therapy group. Accordingly, as already mentioned, no further risk factor analysis was possible. Additionally, this is a retrospective study with short-term follow-up. Therefore, patients might still develop late complications, although it is considered appropriate to report on PJI control after one year of follow-up as survival plateaus after that point [45]. Furthermore, it is possible that patients might have undergone revision surgery elsewhere and the reported revision risk must be considered a low-end estimate. While the study could show that ß-TCP is a safe option as a local carrier, the study did not conduct drainage analysis to further evaluate the, already in-vitro published, release kinetics [17]. As vancomycin was only measured in patients with systemic application, it is not possible to distinguish between isolated, local, and systemic use. Furthermore, successful infection control of hip PJI is multifactorial and, while local antibiotic therapy is an important cornerstone, there are multiple other factors that contribute to its success that cannot be controlled in such a study setting and there are reports with good outcomes that do not use any local antibiotics [8]. Lastly, the authors want to emphasise that, as a single-centre study, the application of local antibiotic carriers was influenced by the decision of the head surgeons and the general treatment principle carried out in the referral centre. This could have led to a selection bias in which cases the application was conducted.

To further support the possible advantage of using local antibiotic-loaded tricalciumphosphate carriers, randomized, multi-centre studies are urgently needed.

## 6. Conclusions

Mouldable collagen–tricalciumphosphate composite bone substitute as a local antibiotic carrier in revision hip arthroplasty appears to be a valid option for local antibiotic delivery without systemic complications. However, the risk of repeat revisions due to recurrent or persisting infection in a comorbid patient cohort is around 17% at short-term follow-up.

## Figures and Tables

**Table 1:** Patient parameters.

Parameter	Patient Data
*n*	79
Operations performed	124
Female (%)	39 (49)
Mean Age (SD; range) (years)	69 (11; 46–94)
Mean BMI (SD; range)	29.8 (7.2; 19–47)
Mean ASA Score (SD; range)	3 (0.2; 1–4)
Mean Previous surgeries (SD; range)	4 (2; 1–9)
Mean surgery time (SD; range) (minutes)	176 (74; 61–486)
Foams used per surgery (SD; range)	1.5 (0.5; 1–3)
Dual-loaded foam applications (%) *	19 (15)

* In 15 cases, two agents in two foams were used—most commonly, one vancomycin and one meropenem application.

**Table 2:** Pathogens.

Pathogen	Cases Detected
*Staphylococcus epidermidis*	25
*Staphylococcus aureus*	11
Other Staphylococci	19
*Escherichia coli*	5
*Streptococcus* spp.	4
*Cutibacterium* spp.	6
*Enterococcus* spp.	6
Other Gram-positive pathogens	3
Gram-negative pathogens	9
*Candida* spp.	6

## Data Availability

The datasets used and/or analysed during the current study are available from the corresponding author on reasonable request.

## Supplementary Materials

The following supporting information can be downloaded at: https://www.mdpi.com/article/10.3390/antibiotics13060510/s1, Video S1: Intraoperative application of a mouldable collagen-tricalciumphosphate.
